# Symptoms and quality of life in patients with chronic obstructive pulmonary disease treated with aclidinium in a real-life setting

**DOI:** 10.3402/ecrj.v3.31232

**Published:** 2016-07-05

**Authors:** Peter Lange, Nina Skavlan Godtfredsen, Beata Olejnicka, Bo-Anders Paradis, Dan Curiac, Sjur Humerfelt, Gunilla Telg, Helene Nordahl Christensen, Magnus Alexander Bitsch, Elisabeth Wreford Andersen, Leif Bjermer

**Affiliations:** 1 Section of Social Medicine, Department of Public Health, Copenhagen University, Copenhagen, Denmark; 2 Respiratory Section, Hvidovre Hospital, Copenhagen University, Copenhagen, Denmark; 3 Airway Inflammation Unit, Department of Experimental Medical Science, Lund University, Lund, Sweden; 4 Department of Internal Medicine, Trelleborg Hospital, Trelleborg, Sweden; 5 Vårdcentralen Näsby, Kristianstad, Sweden; 6 Clinical Trial Center, Gothia Forum, Sahlgrenska University Hospital, Gothenburg, Sweden; 7 Clinic of Allergology and Respiratory Medicine, Oslo, Norway; 8 AstraZeneca Nordic-Baltic, Södertälje, Sweden; 9 Department of Applied Mathematics and Computer Science, Technical University of Denmark, Lyngby, Denmark; 10 Department of Respiratory Medicine and Allergology, Lund University, Lund, Sweden

**Keywords:** patient-reported outcomes, patient satisfaction, dyspnoea, COPD, LAMA, observational study

## Abstract

**Introduction:**

Chronic obstructive pulmonary disease (COPD) is a progressive disease with symptoms that can have a major impact on patients’ physical health. The aim of this study was to evaluate quality of life (QoL), symptom severity and dyspnoea in COPD patients treated with aclidinium up to 24 weeks.

**Methods:**

In this prospective non-interventional multicentre study (198 centres in Sweden, Denmark, and Norway), COPD patients (age ≥40 years) who started treatment with aclidinium (initial therapy, change of treatment, or add-on therapy) could be included. Health-related QoL was obtained by COPD assessment test (CAT). Symptoms were evaluated on a 6-point Likert scale. The modified Medical Research Council (mMRC) Dyspnoea Scale was used as a simple grading system to assess the level of dyspnoea/shortness of breath from0 to 4. Patients on treatment with aclidinium who completed baseline and at least one follow-up visit (week 12 or 24) were included in the study population.

**Results:**

Overall, 1,093 patients were enrolled (mean 69 years, 54% females), one-third had ≥1 exacerbation the year prior to baseline. At enrolment, 48% were LAMA naïve. Mean (standard deviation, SD) CAT score decreased from 16.9 (7.7) at baseline to 14.3 (7.3) at week 24 (*p*<0.01) with a decrease in all individual CAT items (*p*<0.05). Mean difference in morning and night-time symptoms from baseline to week 24 was −0.60 (SD 2.51) and −0.44 (SD 2.48), respectively (both *p*<0.001). Mean (SD) mMRC Dyspnoea Scale changed from 1.6 (1.0) at baseline to 1.5 (1.0) at week 24 (*p*<0.001).

**Conclusion:**

In this observational study of a Nordic real-life COPD population, treatment with aclidinium was associated with a clinically important improvement in QoL and morning and night-time symptoms, most pronounced in the LAMA naïve group. However, there is still room for improvement in the management of symptomatic COPD patients.

Chronic obstructive pulmonary disease (COPD) is a major cause of morbidity and mortality worldwide (1). As the condition progresses, the burden and severity of symptoms increase, leading to physical health limitations and reduced independence in activities of daily living. Current treatment strategies aim to improve symptom control and to reduce the risk of future exacerbations (1). It is recognized that the quality of an effective COPD management should be assessed by parameters such as forced expiratory volume in 1 sec (FEV_1_), and patient-related outcomes such as daily activities, burden of symptoms and health-related quality of life (QoL). The patient-reported questionnaire, COPD assessment test (CAT) (2), has been included in the current COPD treatment guidelines in addition to the disease classification by spirometry (1).

COPD symptoms such as dyspnoea are generally worse in the morning than during the rest of the day, affecting morning routine activities and basic self-care tasks (3). Patients with COPD also commonly experience night-time symptoms that have an impact on their ability to get up in the morning (4). Also, it has been shown that morning symptoms may affect patients’ ability to perform daily life activities throughout the day (5).

Pharmacotherapy for COPD relies primarily on inhaled medications. In a retrospective real-life study of COPD patients in Sweden, it was shown that the introduction of a long-acting inhaled anticholinergic agent (LAMA) and fixed inhaled corticosteroids (ICS)/long-acting β_2_ agonist (LABA) combinations in COPD treatment coincides with a decreased number of COPD exacerbations over a 10-year period (6). For symptomatic patients with low risk of exacerbations, guidelines recommend LAMA or LABA as first-line maintenance treatment (1). In addition to current available LAMAs, aclidinium in the Genuair inhaler is a new LAMA and the only LAMA that is administered twice daily. In clinical studies, it has demonstrated an effective bronchodilation, not only during the day but also at night, resulting in a significant improvement of symptoms and QoL (7–9).

There are limited data available about the effect of aclidinium in a real-life COPD population. The aim of this study was to evaluate QoL, symptom severity (including morning and night-time symptoms), and dyspnoea in COPD patients treated with aclidinium for up to 24 weeks.

## Materials and methods

### Study design and data source

This was a prospective non-interventional multicentre study conducted at 198 primary care and specialist outpatient centres in Sweden, Denmark, and Norway. At baseline (visit 1), sociodemographic data (sex, age, height, and weight) and medical data (smoking status, exacerbations, spirometry including reversibility test, co-morbidities, and concomitant medication) were collected. Data were registered in an electronic data capture system and stored in a secure database managed by the Department of Applied Mathematics and Computer Science, Technical University of Denmark (DTU, Copenhagen, Denmark). Patients were asked to complete a health-related QoL questionnaire at baseline and at the 12 (visit 2) and 24 weeks (visit 3) follow-up visits.

The study was approved by the regional ethics committee in Lund, Sweden (ref. no. 2013/499), the regional committees for medical and health research ethics in Oslo, Norway (ref. no. REK sør-øst 2013/1261), and permission to compile data was granted by Danish data protection agency in Copenhagen, Denmark (ref. no. 2013-41-2236). All patients gave written informed consent to the documentation and processing of their data.

### Study population and treatment

Male and female patients (age ≥40 years) with COPD, who started treatment with aclidinium administered according to specifications in the summary of product characteristics (322 µg aclidinium twice daily), either as initial therapy, change of treatment or as add-on therapy could be included in the study. The decision to initiate aclidinium treatment had to be made prior to the decision to include the patient into the study. The COPD diagnosis was established according to clinical practice and a spirometry, not older than 3 months at inclusion. Patients with pulmonary disease other than COPD, acute COPD exacerbation within 1 month prior to inclusion, and women who were pregnant or breast-feeding were not eligible for inclusion. The patient enrolment was conducted from November 2013 to December 2014.

### Measurements and outcomes

#### Assessments

Health-related QoL was obtained from self-administered patient questionnaires by using the Swedish, Danish, and Norwegian versions of the CAT (2, 10). The CAT comprises eight items each with a scoring range of 0–5. The CAT total score is derived as the sum of responses given in the eight items with a range of 0–40. A minimum clinically important improvement in CAT has been identified to be −2.0 (11).

COPD symptoms were assessed from patient questionnaires according to five indicators: coughing during morning, coughing during night-time, breathlessness during morning, breathlessness during night-time, and quality of sleep. The severity of these symptoms was evaluated on a 6-point Likert scales rated from 0=‘no symptoms’ to 5=‘very severe symptoms’ and from 0=‘very bad sleep’ to 5=‘very good sleep’ (12).

The modified Medical Research Council (mMRC) Dyspnoea Scale was used as a simple grading system to assess the level of dyspnoea/shortness of breath in five categories from 0 to 4 (1).

At each study visit, patients reported the presence of any adverse events between visits. If judged causal to aclidinium by the investigator, the event was reported as an adverse drug reaction (ADR).

#### Other measurements

Classification of patients according to GOLD A–D criteria was based on the GOLD spirometry classifications of the severity of airflow limitation, exacerbation history in the past year, and the patient's symptoms, using CAT (1).

Smoking status was defined as current smoker, ex-smoker, or never smoker.

The BMI was defined as the body weight in kilograms divided by the square of height in meters.

BMI categories: underweight ≤18.5; normal weight=18.5–24.9; overweight=25–29.9; obesity=BMI of 30 or greater.

Lung function was defined as the percentage of FEV_1_ predicted value and should be no older than 3 months prior to visit 1.

Co-morbidity was defined according to the following selected chronic diseases: current treatment of depression, diabetes, heart failure, ischemic heart disease, hypertension, osteoporosis, gastro-oesophageal reflux, and disease of the musculoskeletal system (or other diseases inhibiting walking).

Concomitant medications to be taken together with aclidinium were categorized as follows: short-acting β_2_-agonist (SABA), LABA, ICS, oral corticosteroids (OCS), phosphodiesterase (PDE) 4 inhibitor, fixed ICS/LABA combination, fixed SABA/short-acting muscarinic antagonist (SAMA), other concomitant medication, or no concomitant medication.

A combined variable regarding prior medication was constructed based on information about treatment with LAMA prior to inclusion (yes/no) and concomitant maintenance medication to be taken together with aclidinium (LABA, ICS, PDE4, and/or fixed ICS/LABA combination). The patients were categorized into the following four subgroups: LAMA naïve without other maintenance therapies, LAMA naïve with other maintenance therapies, LAMA non-naïve without other maintenance therapies, and LAMA non-naïve with other maintenance therapies. Maintenance treatment included ICS, LABA, and LAMA.

Patient-reported satisfaction with the inhaler and handling of the Genuair device was collected.

### Statistical analysis

The study population was defined as all patients who completed the baseline visit and at least one follow-up visit (week 12 or 24) and continued on treatment with aclidinium during the defined study period. Continuous and nominal variables were described using standard statistical measures, that is, number of observations, mean, and standard deviation. All categorical variables were summarized with absolute and relative frequencies.

Baseline characteristics were compared over the four prior medication groups using one-way ANOVA tests for continuous variables and chi-squared test for categorical variables (and in cases where the expected numbers were below five, Fisher's exact test). The paired t-test was used to compare the total CAT score and symptoms from baseline to week 12 or baseline to week 24 to take into account that the same group of patients were followed through the three visits. Similarly, for categorical variables McNemar's chi-squared test was used to compare baseline to week 12 or 24.

An ANCOVA model was fitted to estimate the expected changes in total CAT score for the four prior medication groups from baseline to week 12 or 24 while taking baseline CAT score into account. Finally, a multivariate logistic regression model was used to estimate the odds of reaching a clinically important difference of at least two units on total CAT score from baseline to week 12. This model included the covariates: baseline CAT score, age, sex, current smoker (yes/no), BMI group (underweight, normal, overweight/obese), FEV_1_ ≥50% of the predicted value (yes/no), heart failure or ischemic disease (yes/no), and prior medication.

## Results

### Patient flow

**Fig. 1:**
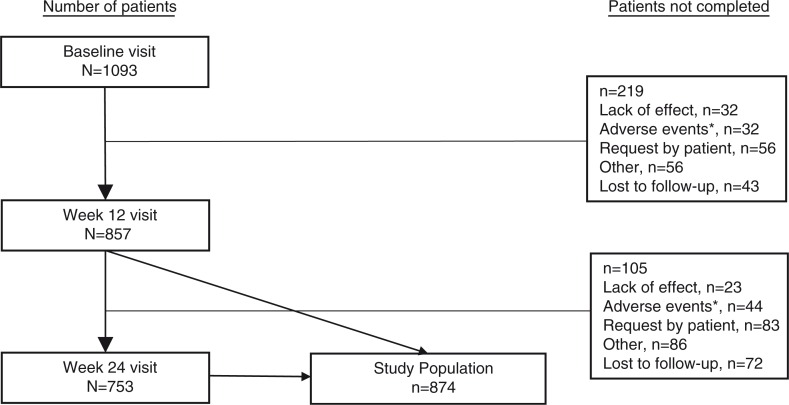
Patient flowchart.

A total of 1,093 patients were enrolled across the 198 study sites in Sweden, Denmark, and Norway. Follow-up visits were completed for 78% (*n=*857) of the enrolled patients at week 12 and 69% (*n*=753) at week 24 (Fig. 1). Overall, 80% (*n*=874) completed the baseline visit and at least one follow-up visit, and thus comprised the study population.

### Baseline characteristics

Mean age was 69 years and 54% were females (Table 1). The majority had a history of smoking (96% current or ex-smokers) and more than half of the patients (56%) were overweight or obese. Baseline lung function measured within 3 months prior to study enrolment showed a mean FEV_1_ percentage of predicted normal of 55%. One-third of the patients had experienced one or more exacerbations during the year prior to baseline. The majority of the patients were classified as either GOLD B (42%) or GOLD D (42%). The leading co-morbidity was hypertension (37%) followed by ischemic disease (12%) and diabetes mellitus (11%) (Table 1). No differences were seen regarding baseline patient characteristics between the patients included in the study population and the patients who were lost to follow-up (results not shown).

**Table 1:** Demographic and clinical characteristics at baseline visit among participants who completed baseline and at least one follow-up visit (study population and subgroups based on prior medication)

Characteristics at baseline visit	Study population (*N*=874)	LAMA naïve without maintenance (*N*=245)	LAMA naïve with maintenance (*N*=172)	LAMA non-naïve without maintenance (*N*=112)	LAMA non-naïve with maintenance (*N*=345)	*p*^a^
Country, *n* (%)						<0.001
Sweden	497 (57)	100 (41)	79 (46)	65 (58)	253 (73)	
Denmark	292 (33)	124 (51)	71 (41)	33 (29)	64 (19)	
Norway	85 (10)	21 (9)	22 (13)	14 (12)	28 (8)	
Gender, *n* (%)						0.069
Men	398 (46)	120 (49)	89 (52)	47 (42)	142 (41)	
Age (years), mean (SD)	69.3 (9.1)	67.8 (9)	68.7 (10)	68.8 (9.4)	70.9 (8.4)	<0.001
Age categories (years), *n* (%)						0.002
40–49	25 (3)	10 (4)	9 (5)	1 (1)	5 (1)	
50–59	105 (12)	35 (14)	23 (13)	20 (18)	27 (8)	
60–69	298 (34)	93 (38)	55 (32)	42 (38)	108 (31)	
70–79	332 (38)	85 (35)	59 (34)	36 (32)	152 (44)	
> 80	114 (13)	22 (9)	26 (15)	13 (12)	53 (15)	
Smoking status, *n* (%)						<0.001
Current smoker	314 (36)	127 (52)	58 (34)	41 (37)	88 (26)	
Ex-smoker	527 (60)	111 (45)	104 (60)	65 (58)	247 (72)	
Never smoker	33 (4)	7 (3)	10 (6)	6 (5)	10 (3)	
BMI, mean (SD)	26.2 (5.2)	26.3 (5.5)	26.4 (4.8)	26.6 (5.7	25.9 (5.1)	0.542
BMI, *n* (%)						0.812
Underweight	48 (5)	12 (5)	6 (3)	7 (6)	23 (7)	
Normal weight	342 (39)	100 (41)	64 (37)	38 (34)	140 (41)	
Overweight	287 (33)	80 (33)	59 (34)	39 (35)	109 (32)	
Obesity	197 (23)	53 (22)	43 (25)	28 (25)	73 (21)	
FEV1 (% pred^b^), mean (SD)	54.9 (16.3)	61.5 (14.6)	56.2 (16.2)	59.0 (14.9)	48.3 (15.5)	<0.001
FEV1 (% pred^b^), n (%)						<0.001
< 30%	59 (7)	4 (2)	8 (5)	4 (4)	43 (12)	
30 to <50%	261 (30)	47 (19)	48 (28)	24 (21)	142 (41)	
50 to <80%	494 (57)	169 (69)	104 (60)	73 (65)	148 (43)	
> 80%	60 (7)	25 (10)	12 (7)	11 (10)	12 (3)	
GOLD A–D,^c^*n* (%)						<0.001
A	87 (10)	39 (16)	11 (6)	14 (12)	23 (7)	
B	368 (42)	137 (56)	79 (46)	53 (47)	99 (29)	
C	48 (5)	8 (3)	11 (6)	9 (8)	20 (6)	
D	371 (42)	61 (25)	71 (41)	36 (32)	203 (59)	
Exacerbations 1 year prior to baseline, *n* (%)						<0.001
0	537 (61)	185 (76)	98 (57)	68 (61)	186 (54)	
1	205 (23)	45 (18)	48 (28)	27 (24)	85 (25)	
2	78 (9)	11 (4)	14 (8)	12 (11)	41 (12)	
≥ 3	54 (6)	4 (2)	12 (7)	5 (4)	33 (10)	
Co-morbidities, *n* (%)						0.129
Depression	66 (8)	19 (9)	8 (5)	10 (9)	29 (7)	
Diabetes	98 (11)	27 (12)	23 (14)	17 (15)	31 (8)	
CV (heart failure or ischemic disease)	154 (18)	29 (13)	31 (19)	17 (15)	77 (19)	
Hypertension	330 (38)	92 (43)	61 (37)	42 (36)	135 (34)	
Osteoporosis	76 (9)	13 (6)	13 (8)	10 (9)	40 (10)	
Gastro-oesophageal reflux	75 (9)	11 (5)	11 (7)	11 (9)	42 (11)	
Disease of the musculoskeletal system	97 (11)	25 (12)	17 (10)	10 (9)	45 (11)	
Baseline concomitant medication, *n* (%)						
SABA	333 (27)	62 (25)	70 (27)	49 (41)	152 (26)	
LABA	126 (10)	0 (0)	53 (20)	0 (0)	73 (12)	
ICS	57 (5)	0 (0)	23 (9)	0 (0)	34 (6)	
Oral steroids	14 (1)	3 (1)	0 (0)	5 (4)	6 (1)	
PDE4 inhibitor	16 (1)	0 (0)	1 (0)	0 (0)	15 (3)	
Fixed ICS/LABA combination	392 (32)	0 (0)	107 (41)	0 (0)	285 (48)	
Fixed SABA/SAMA combination	10 (1)	1 (0)	0 (0)	2 (2)	7 (1)	
Other	39 (3)	7 (3)	7 (3)	6 (5)	19 (3)	
No concomitant medication	232 (19)	175 (71)	0 (0)	57 (48)	0 (0)	
Switch from other LAMA, *n* (%)						
Yes	457 (52)	0 (0)	0 (0)	112 (100)	345 (100)	

a *p*-Value for difference in prior medication by chi-squared test (categorical) and ANOVA (continuous).

b Percentage of forced expiratory volume in 1 sec (FEV_1_) predicted value.

c GOLD spirometry classifications based on the severity of airflow limitation, exacerbation history in the past year, and the patient's symptoms.

During enrolment, 52% (*n*=457) of the patients were switched from another LAMA medication and 48% (*n*=417) were new initiated to aclidinium. The proportion of patients in each of the four subgroups based on prior medication was 20% LAMA naïve with maintenance, 28% LAMA naïve without maintenance, 39% LAMA non-naïve with maintenance, and 13% LAMA non-naïve without maintenance (Table 1). Compared to participants without maintenance medication, those on maintenance medication had a lower FEV_1_ in % of predicted value and more of them belonged to GOLD D group (Table 1). Two-thirds of the patients used aclidinium as add-on therapy; the most frequent maintenance medication was fixed ICS/LABA combinations (32%), followed by LABA (10%). In addition, 27% of the patients used SABA.

### Health-related QoL (CAT)

**Fig. 2:**
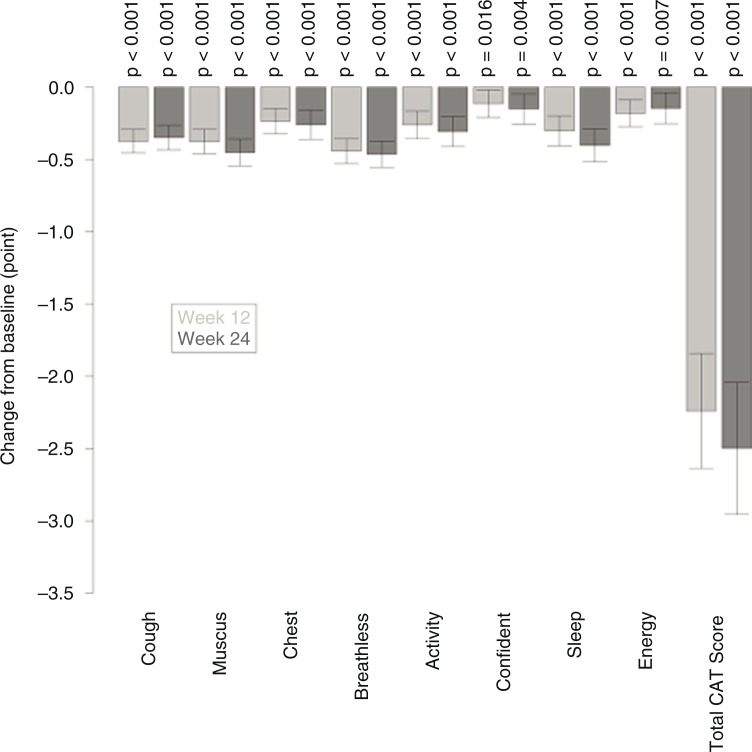
Change in individual CAT item scores and total CAT score from baseline to week 12 (light grey bars) and from baseline to week 24 (dark grey bars). Sample size: *N*=774 (week 12) and *N* = 679 (week 24). ‡*p*<0.01 for baseline versus follow-up (weeks 12 and 24); †*p*<0.05 for baseline versus follow-up (weeks 12 and 24).

The mean (SD) CAT total score changed significantly from 16.9 (7.7) at baseline to 14.6 (7.3) points at week 12, and to 14.3 (7.3) at week 24 (*p*<0.01 for both). Figure 2 shows the changes in the total score and in the individual CAT item scores. All individual CAT item scores decreased significantly from baseline (*p*<0.05; Fig. 2) with the largest mean improvement in the item ‘breathless when walking up a hill or one flight of stairs’.

A clinically relevant improvement in the total CAT score (minimal clinically important difference by at least two points) was observed in 55% for the patients with complete CAT data available. The estimated improvement in total CAT score was highest in the LAMA naïve patients without maintenance treatment; while controlling for baseline CAT score, the mean change at week 12 was −3.5 (95% confidence interval [CI] −4.2 to −2.9) and at week 24 −3.8 (95% CI −4.6 to −3.1) (Table 2). Looking at the pairwise comparisons between the four groups at week 12, the improvement in the LAMA naïve without maintenance treatment was significantly higher than in the two LAMA non-naïve groups (with maintenance *p*<0.01, without maintenance *p=*0.04 [data not shown]).

**Table 2:** Estimated change in CAT total score from baseline to week 12 and from baseline to week 24 adjusting for baseline CAT score with test for effect of prior medication

	Change from baseline to week 12		Change from baseline to week 24	
		
Prior medication	Estimated change (95% CI)	*p*	Estimated change (95% CI)	*p*
LAMA naive without maintenance	−3.54 (−4.21; −2.86)	0.0296	−3.82 (−4.58; −3.05)	0.0410
LAMA naive with maintenance	−2.75 (−3.55; −1.95)		−3.31 (−4.23; −2.38)	
LAMA non-naive without maintenance	−1.90 (−2.89; −0.90)		−2.85 (−3.95; −1.74)	
LAMA non-naive with maintenance	−1.39 (−1.96; −0.83)		−1.41 (−2.04; −0.78)	

Sample size: *n*=774 (week 12) and *n*=679 (week 24).

### Predictors of improvement in CAT total score

The results from the multivariate logistic regression (Table 3) shows that prior medication was the strongest predictor of reaching a clinically relevant improvement of at least two units on the total CAT score from baseline to week 12 (*p*<0.01). Compared to the LAMA non-naïve with maintenance, the LAMA naïve groups have significantly higher odds of improving when adjusting for baseline CAT score, sex, age, smoking status, BMI group, FEV_1_, and cardio vascular disease (odds ratio [OR] 1.9 (95% CI 1.3–2.9) for naïve with maintenance and OR 1.8 [95% CI 1.3–2.7] without maintenance). Also, higher baseline CAT score and FEV_1_>50% of predicted value were significant predictors of clinically relevant improvement in CAT.

**Table 3:** Predictors of improvement in CAT total score (at least two points) from baseline to week 12 (multivariate logistic regression)

	CAT total score improvement (at least two points)	
		
	OR (95% CI)	*p*
CAT baseline score^a^	1.10 (1.08; 1.13)	<0.01
Women	1.26 (0.95; 1.69)	0.11
Men	(ref.)	
Age (years)	1.00 (0.98; 1.02)	0.87
Current smoker (baseline)	0.87 (0.63; −1.20)	0.41
Not current smoker (baseline)	(ref.)	
BMI (baseline)		
Underweight	1.39 (0.72; 2.74)	0.54
Normal	(ref.)	
Overweight or obese	1.12 (0.83; 1.52)	
FEV1 ≥50% (baseline)	1.57 (1.14; 2.16)	0.01
FEV1 <50% (baseline)	(ref.)	
CV co-morbidity^b^ (baseline)	0.83 (0.57; 1.23)	0.36
No CV co-morbidity (baseline)	(ref.)	
Prior medication (baseline)		
LAMA naive without maintenance	1.84 (1.26; 2.71)	<0.01
LAMA naive with maintenance	1.95 (1.30; 2.95)	
LAMA non-naive without maintenance	1.19 (0.75; 1.91)	
LAMA non-naive with maintenance	(ref.)	

a COPD assessment test measured at baseline visit.

b CV comorbidity = heart failure or ischemic disease.

### Severity of morning and night-time symptoms

**Fig. 3:**
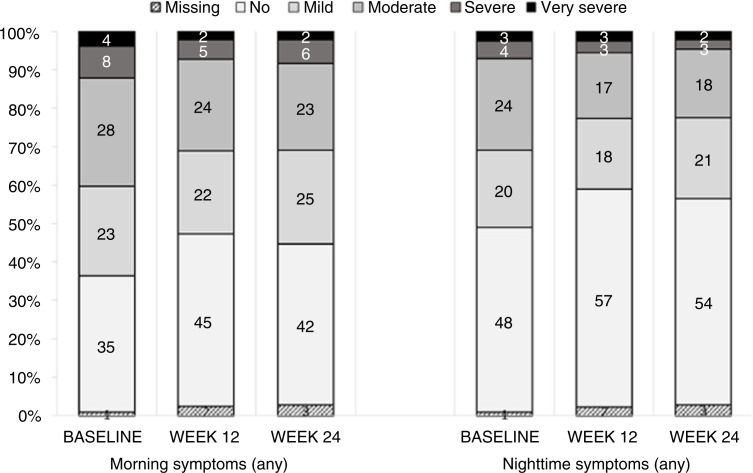
Prevalence of morning and night-time COPD symptoms (any) at baseline, week 12, and week 24.

The proportion of patients with no morning symptoms changed from 35% at baseline to 45% at week 12, and to 42% at week 24 (Fig. 3). Moderate-to-very severe morning symptoms were reported by 40% of the patients at baseline and by 31% after 24 weeks of follow-up (Fig. 3). The proportion of patients with no night-time symptoms was 48% at baseline, 57% at week 12, and 54% at week 24 (Fig. 3). Moderate-to-very severe night-time symptoms were observed for 31% of the patients at baseline and for 23% after 24-week follow-up (Fig. 3).

**Fig. 4:**
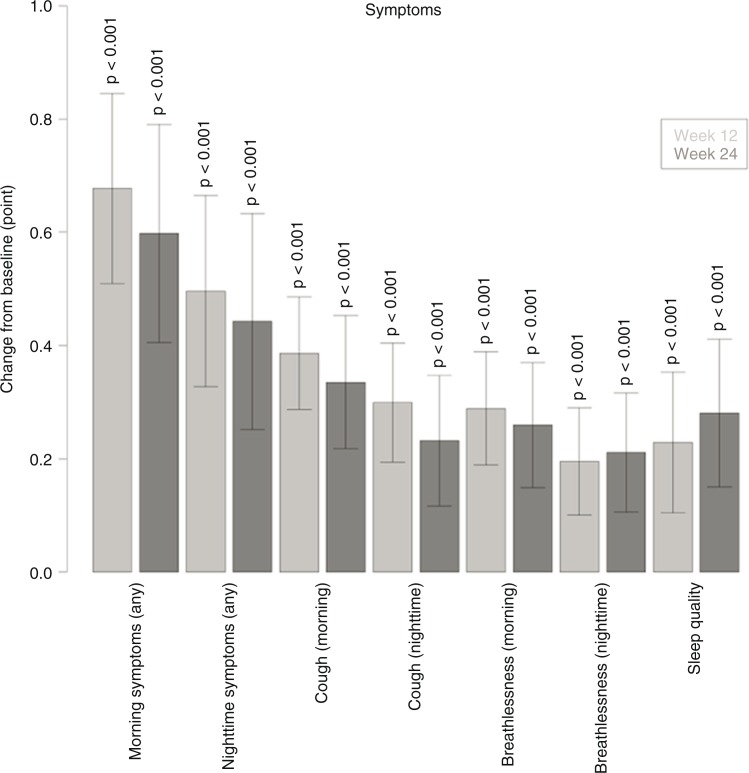
Change in severity of morning and night-time symptoms (any symptom, cough, breathlessness, sleep quality) from baseline to week 12 (light grey bars) and from baseline to week 24 (dark grey bars). Sample size: *N*=797. ‡*p*<0.001 for baseline versus follow-up (weeks 12 and 24).

There was a statistically significant improvement in morning and night-time symptoms (any symptom, cough, breathlessness, or/and sleep quality) (*p*<0.001; Fig. 4). The largest improvement was found for ‘morning symptoms (any)’: week 12: mean difference of −0.68 (SD 2.34) and week 24 mean difference of −0.60 (SD 2.51). Further, a statistically significant improvement in all subgroups except LAMA non-naïve patients without maintenance was observed for morning and night-time symptoms (Table 4).

**Table 4:** Subgroup analysis of change in morning symptoms, night-time symptoms, and mMRC Dyspnoea Scale from baseline versus week 12 and baseline versus week 24 according to prior medication

	Prior medication			
	
	LAMA naive without maintenance	LAMA naive with maintenance	LAMA non-naive without maintenance	LAMA non-naive with maintenance
	
	Difference in means (SD)	Difference in means (SD)	Difference in means (SD)	Difference in means (SD)
Change from baseline vs. week 12				
Morning symptoms (any)	−0.9 (2.3)^a^	−0.8 (2.3)^a^	−0.5 (2.5)^a^	−0.6 (2.0)^a^
Night-time symptoms (any)	−0.8 (2.2)^a^	−0.6 (2.4)^a^	−0.3 (2.4)	−0.5 (2.3)^b^
mMRC dyspnoea grade	−0.0 (0.9)	−0.3 (0.9)^a^	−0.1 (0.9)	−0.3 (0.9)^a^
Change from baseline vs. week 24				
Morning symptoms (any)	−0.9 (2.5)^a^	−0.8 (2.4)^a^	−0.3 (2.6)	−0.7 (2.2)^a^
Night-time symptoms (any)	−0.8 (2.3)^a^	−0.5 (2.5)^a^	−0.2 (2.7)	−0.6 (2.2)^b^
mMRC dyspnoea grade	−0.2 (0.8)^b^	−0.3 (0.9)^a^	−0.1 (0.9)	−0.4 (0.8)^a^

Sample size: *n*=774 (week 12) and *n*=679 (week 24).

a *p*<0.01 for baseline versus follow-up (weeks 12 and 24)

b *p*<0.05 for baseline versus follow-up (weeks 12 and 24).

### Breathlessness (the mMRC Dyspnoea Scale)

The mean (SD) mMRC Dyspnoea Scale changed significantly (*p*<0.001) from 1.6 (1.0) at baseline to 1.5 (1.0) at weeks 12 and 24 (Table 5). The proportion of patients with an mMRC grade ≥2 changed from 52% at baseline to 45% at week 12 and 42% at week 24 (Table 5).

**Table 5:** Proportion of patients (%) at each mMRC dyspnoea grade from 0 to 4 at baseline (*N=*797), week 12 (*N=*774), and week 24 (*N*=679)

	Baseline	Week 12	*p*	Week 24	*p*
mMRC grade, mean (SD)	1.6 (1.0)	1.5 (1.0)		1.5 (1.0)	
Difference in means (SD)		−0.2 (0.9)	<0.001	−0.2 (0.9)	<0.001
mMRC grade, *n* (%)					
Grade 0	98 (12.3)	122 (15.8)		109 (16.1)	
Grade 1	274 (34.4)	284 (36.7)		258 (38.0)	
Grade 2	254 (31.9)	215 (27.8)		179 (26.4)	
Grade 3	135 (16.9)	112 (14.5)		92 (13.5)	
Grade 4	29 (3.6)	19 (2.5)		16 (2.4)	
Missing	7 (0.9)	22 (2.8)		25 (3.7)	
mMRC grade ≥ 2, *n* (%)	418 (52.4)	345 (44.6)	<0.001	287 (42.3)	<0.001

Respondents with ‘missing’ are not included in calculating *p* values.

### Adverse drug reactions

During this 6-month study, 46 patients (4%) reported in total 102 ADRs, whereof 29 were reported as serious adverse events. Overall, 33 patients discontinued study drug due to ADR. The most commonly reported ADR was dysphonia (0.9%), unpleasant product taste (0.7%), headache (0.7%), dyspnoea (0.5%), and nausea (0.5%). All other ADRs reported had an incidence of <0.5%. One serious adverse event was fatal (cardiac arrest), however, without reported drug causality.

### Patient handling and satisfaction of the Genuair device

Overall, 95% of the patients found the Genuair device easy or very easy to use and 68% of the patients were satisfied or very satisfied with the device.

## Discussion

In this real-life COPD population recruited from general practice and outpatient specialist care, including both LAMA-naïve patients and LAMA switchers, both with and without concurrent COPD maintenance medications, treatment with aclidinium during 24 weeks was associated with a significant improvement in both QoL and in early morning and night-time COPD symptoms. The most pronounced improvement was observed after 12 weeks, whereas the difference between weeks 12 and 24 was smaller. This indicates an effect within 12 weeks, with the greatest improvement seen in the LAMA-naïve patients. However, approximately one out of four patients still experienced moderate-to-very severe morning and night-time symptoms at follow-up, indicating suboptimal symptom control.

Overall, the addition of a LAMA to ICS and/or LABA treatment was associated with a beneficial effect, but the most important predictor of improvement in CAT score was being LAMA naïve.

The beneficial effect of adding a LAMA to ICS/LABA treatment has previously been reported in randomized clinical trials (13, 14). In the present study, almost half of the patients were LAMA naïve at baseline, fewer than in previous studies (8, 15). In the recently published Austrian real-life study with a similar design (*n*=795, 12-week follow-up, mean age 64 years, 44% female), three out of four patients were LAMA naïve at baseline (16). The improvements in CAT, mMRC Dyspnoea Scale, and symptoms observed in that study were greater than what was seen in the present study, which may be explained by the slightly younger study population and the fact that the majority of the patients in that study were LAMA naïve.

The literature suggests that COPD symptoms are worst during morning, with four out of five COPD patients experiencing shortness of breath in the morning (17). Night-time symptoms are also prevalent and have been associated with worsening of COPD severity (18), risk of future exacerbations (19), poor QoL (20, 21), increased anxiety and depression (22), and mortality (23, 24). Furthermore, it has been shown that patients commonly take their medication too late in the morning to have an effect on morning symptoms (17). The LAMAs available for the treatment of COPD have a once-daily regimen, except for aclidinium which is administered twice daily. Patient preference regarding dosing regimens varies, as shown in a recent study on asthma and COPD patients where only half of the patients actually preferred the once-daily regimen (25). In addition, significant improvements in night-time symptom severity were shown for aclidinium but not for tiotropium compared to placebo (8). Also, a mean FEV_1_ below baseline was reported for tiotropium during a prolonged period of the night compared to aclidinium (8). For the LAMA-naïve patients in the present study, a positive effect on health status would likely be observed by adding any LAMA. For patients switched from another LAMA to aclidinium, the positive effect on symptoms may be explained by the twice daily dosing of aclidinium, potentially increased by a placebo effect due to study participation.

The main limitation of the present study is the observational design with the absence of a control group. The association found may have been affected by other factors impacting patient-reported outcomes, such as participation in a study. No information regarding the reason for the patients’ visit to the physician during which aclidinium was initiated was collected (scheduled follow-up visit or a visit due to disease deterioration) and patient adherence to treatment was not monitored. Furthermore, two-thirds of the patients used aclidinium as add-on therapy and it cannot be ignored that the use of concomitant maintenance medications for COPD may have influenced the results. Bias due to the unknown disease severity of the patients lost to follow-up cannot be excluded; however, a comparison of patient characteristics between the study population and the lost to follow-up population showed similar groups at baseline. Approximately, 5% of the recruited patients were excluded from the study before the week 24 visit due to lack of medication effect, and as these patients are not included in the study population, our results are slightly skewed towards favouring the effect of aclidinium.

However, as most pivotal pharmacological trials exclude patients suffering from significant co-morbidities, the real-life character of the present study expands currently available knowledge, which is derived almost exclusively from controlled randomized trials, performed in highly selected patients by narrow inclusion criteria, resulting in low external validity.

The safety data obtained in this study are consistent with the safety and tolerability data reported in other studies (7, 8, 16).

## Conclusion

In this observational study of a Nordic real-life COPD population recruited from general practice and outpatient specialist care, we found that that treatment with aclidinium was associated with a significant and clinically important improvement in QoL and in morning and night-time symptoms after 12 weeks, primarily in LAMA-naïve patients but also in non-naïve patients. However, as a proportion of patients still experienced moderate-to-very severe morning and night-time symptoms at study end, there is still room for improvement in the everyday management of symptomatic patients with COPD.
